# ZAG (Zinc-Alpha 2 Glycoprotein) Serum Levels in Girls with Anorexia Nervosa

**DOI:** 10.3390/jcm12134245

**Published:** 2023-06-24

**Authors:** Jarzumbek Anna, Świętochowska Elżbieta, Mizgała-Izworska Elżbieta, Gołąb-Jenerał Katarzyna, Bąk-Drabik Katarzyna, Ziora Katarzyna

**Affiliations:** 1 Department of Paediatrics, Faculty of Medical Sciences in Zabrze, Medical University of Silesia, 41-800 Zabrze, Poland; 2 Department of Medical and Molecular Biology, Faculty of Medical Sciences in Zabrze, Medical University of Silesia in Katowice, 41-808 Zabrze, Poland; 3 Department of Family Medicine, Faculty of Medical Sciences in Zabrze, Medical University of Silesia in Katowice, 41-808 Zabrze, Poland

**Keywords:** anorexia nervosa, obesity, lipolysis

## Abstract

The objectives of the study were: (1) the evaluation of the blood serum concentration of ZAG (Zinc-alpha 2 Glycoprotein) in girls with anorexia nervosa, as well as in girls with simple obesity and healthy girls; and (2) the valuation of the relationship between the blood level of ZAG and the duration of AN and anthropometric parameters, parameters of the lipid and carbohydrate metabolism, thyroid hormones, and cortisol in the blood in all study subjects. Materials and methods: The study covered 87 girls (aged 11–17.9 years). The studied group (AN) contained 30 girls suffering from anorexia nervosa, and the control groups contained 30 healthy girls (H) and 27 girls with simple obesity (OB), respectively. Results: The mean concentration of ZAG in the blood serum in the AN group was significantly higher than in the OB and H groups. Accumulatively, the level of ZAG in the entire studied group correlated negatively with the parameters of their nutritional status. The mean concentrations of ZAG in the entire group correlated positively with the concentrations of HDL and cortisol and negatively with insulin, HOMA-IR, triglycerides, and hsCRP. Conclusions: The higher blood concentrations of ZAG in girls with AN compared to healthy subjects seemed to constitute a secondary adaptation mechanism in response to the undernourishment status. ZAG blood concentration values correlated negatively with body mass, BMI, Cole’s index, the level of insulin, and the HOMA-IR score, whereas they correlated positively with the level of cortisol. Increased ZAG levels in AN patients may result from increased levels of cortisol, manifesting in malfunction along the hypothalamic–pituitary–adrenal axis, which in effect can promote body weight loss.

## 1. Introduction

Zinc-*α*2-Glycoprotein (ZAG) is a soluble, glycosylated protein that has been identified in the secretory epithelium of numerous body organs, including the prostate, nipples, liver, pancreas, intestines, and skeletal muscles. In the case of many pathological processes associated with cachexia, such as heart failure, chronic kidney disease, and neoplastic disease, ZAG concentrations in the blood serum increase 2–7 times above their normal limits [1].

It has been demonstrated in animals that ZAG exerts some effects on the metabolism of the adipose tissue through the induction of the lipolysis process, predominantly through the *β*3-adrenergic receptor and, to a lesser extent, through the *β*2 receptor. Their activation increases the intracellular concentrations of cyclic adenosine monophosphate (cAMP), resulting in the hydrolysis of triglycerides and the release of free fatty acids. This effect is lost after the addition of the *β*3-adrenergic receptor antagonist. In addition, ZAG, besides its function associated with lipid mobilisation, demonstrates another activity, consisting in an enhanced use of substrates after their breakdown and intensifying oxidation processes in the mitochondria of brown adipose tissue (BAT) cells. It is also associated with the intensified expression of uncoupling proteins (UCPs): (UCP-1 in the brown adipose tissue and UCP-2 in the miotubes of mice). These proteins mediate the penetration of protons through the internal mitochondrial membrane, weakening the coupling of respiration and ADP phosphorylation. This activity has also been observed in a mechanism dependent on the stimulation of the *β*3-adrenergic receptor [2,3]. In humans, the presence of both ZAG mRNA and ZAG itself has been demonstrated in subcutaneous adipose tissue (SAT) and visceral adipose tissue (VAT). Studies carried out on human preadipocyte cultures have demonstrated that ZAG expression increases under the influence of glycocorticosteroids and through the stimulation of the receptors activated by gamma peroxisome proliferators (PPAR-*γ*). 

Rosiglitazone, an agonist of peroxisome proliferator-activated receptor gamma, induced a three-fold increase in ZAG mRNA concentration in an adipocyte culture, whereas the alpha tumour necrosis factor (TNF-*α*) reduced that concentration four times [2].

Anorexia nervosa (AN), classified as an eating disorder (ED), is the subject of shared interests in both psychiatric and somatic medicine. Many studies assessing proteins and cytokines have proved that the role of immunological factors in anorexia nervosa is significant. In many situations, the attempts to find out whether increased or reduced levels of certain molecules constitute the foundation of the disease or whether they constitute an adaptation factor in the course of cachexia are simply futile. 

The available literature from around the world describes the relationship between an increased level of ZAG and cachexia in the course of diseases such as chronic circulatory insufficiency, chronic kidney disease, and cancer. More and more emphasis is placed on the assessment of this protein’s concentrations in obese patients, where it is suggested to be an obesity-promoting factor through lipolysis suppression [4,5]. There are no sufficient reports describing the levels of this protein in patients suffering from anorexia nervosa. 

Therefore, it seemed very interesting to assay the ZAG protein concentrations in the blood serum of girls with AN, as the role of this protein in the process of cachexia, predominantly in the course of cancer, is becoming increasingly well understood and established. The scientific reports evaluating this protein in the context of AN are incredibly scarce, and the available publications refer mainly to adult populations. 

The objectives of this study were: (1) the evaluation of blood serum ZAG (zinc-alpha 2 Glycoprotein) concentrations in girls suffering from anorexia nervosa and the analysis of its potential importance in AN; (2) the comparison of serum ZAG concentrations between girls with anorexia, girls with simple obesity, and healthy girls with a normal body weight; and (3) the evaluation of the relationships between ZAG blood levels, AN duration, anthropometric parameters, the parameters of lipid and carbohydrate metabolism, thyroid hormones, and cortisol levels in the blood in all the study subjects. 

## 2. Materials

### 2.1. Characteristic Features of the Girls Involved in the Study

The study involved eighty-seven (87) girls, aged 11–17.9 years (mean age: 15.2 ± 0.4), subjected to simultaneous assays of MIC-1 (Macrophage Inhibitory Cytokine-1) concentrations in the blood serum [6]. 

Similarly to the previous study, the study group (AN) consisted of 30 girls, aged 13–17.3 years (mean age: 15.64 ± 0.41 years), suffering from a restrictive form of anorexia nervosa, who had been diagnosed according to the DSM-5 criteria during hospitalisation in the Department of Paediatrics [7].

### 2.2. Criteria for Inclusion in the AN Group

- A diagnosis of the restrictive form of anorexia nervosa according to DSM-V criteria;
- Age 11–18 years;
- Written informed consent obtained from the parents/legal guardians of the girls involved to allow them to participate in the study;
- Written informed consent to participation in the study obtained from the study subject aged ≥16 years.

### 2.3. Criteria for Exclusion from the AN Group

- The purgative form of anorexia nervosa;
- Poor health condition preventing blood sampling, e.g., dehydration or vomiting;
- Abnormal functional parameters and indices of the liver and kidneys;
- Acute infection at the time of the study or during the previous three months before the study onset;
- Pharmacotherapy administered immediately before the study onset;
- The coexistence of another medical condition that may have led to cachexia.

The first control group consisted of 30 healthy girls (H), aged 11.2–17.9 years (mean age: 16.17 ± 0.54 years) with a normal body weight, selected from a collection of volunteers who were students of secondary schools in the junior and senior cycle.

### 2.4. Criteria for Inclusion in Group H

- Normal body weight and BMI, according to the applicable standards for sex and age in the Polish population (Palczewska et al.).
- Age 11–18 years;
- Written informed consent obtained from the parents/legal guardians of the girls involved to allow them to participate in the study;
- Written informed consent to participation in the study obtained from study subject aged ≥16 years.

### 2.5. Criteria for Exclusion from Group H

- Chronic medical conditions;
- Menstruation disorders;
- Acute infectious conditions during the three months before the study onset;
- Pharmacotherapy during the month before the study onset;
- Adherence to various dieting methods during the three months before the study onset or a history of eating disorders.

The second control group consisted of 27 girls with simple obesity (OB), aged 11–17 years (mean age: 13.73 ± 0.82 years), all of them being patients of the Endocrine Outpatient Clinic. 

### 2.6. Criteria for Inclusion in the OT Group

- A diagnosis of simple obesity with reference to the centile grids for BMI, according to Palczewska and Niedźwiedzka (BMI > 97th percentile for sex and age, and BMI SDS > 2 standard deviations);
- Age 11–18 years;
- Written informed consent obtained from the parents/legal guardians of the girls involved to allow them to participate in the study;
- Written informed consent to participation in the study obtained from study subject aged ≥16 years.

### 2.7. Criteria for Exclusiom from Group OT

- Chronic medical conditions other than obesity;
- Genetic syndromes and hormonal disorders concomitant with obesity;
- Menstruation disorders;
- Acute infectious conditions during the three months before the study onset;
- Pharmacotherapy during the month before the study onset;
- Adherence to various dieting methods during the three months before the study onset or a history of eating disorders.

## 3. Methods

### 3.1. Interview, Physical Examination, and Anthropometric Measurements

All the study subjects underwent a detailed interview and physical examination. None of the girls from the control groups suffered from chronic diseases or acute infections during the month before the study starting point. None of them had any eating disorders in their history. 

The girls’ body weights were measured by means of medical scales and their body heights by means of a stadiometer. The obtained values were used as an input in the calculation of the body mass index (BMI) (following the formula: BMI = body mass (kg)/height (m)^2^). These values were also expressed as standard deviation scores (SDSs) from the mean values for age and gender, according to Palczewska et al. [8]. 

In the AN group, the examinations were carried out prior to the therapy onset, during the first days of hospitalisation and after the evaluation of patient somatic condition by a paediatrician; basic lab tests (CBC, concentrations of bilirubin, aminotransferases, creatinine, urea, and total protein in serum); and a mental state test by a clinical psychologist and a psychiatrist.

In menstruating girls from the control groups, the examinations were carried out in the follicular phase. 

The mean body height of the girls with anorexia nervosa was 164.22 ± 2.64 cm (151–182.3 cm), the mean maximum body weight prior to the disease was 58.98 ± 4.35 kg (36.0–82.0 kg), and the mean body weight loss since the disease onset was 15.92 ± 2.77 kg (5.3–33.7 kg) (see Table 1). The mean illness duration from the beginning of intense dieting to hospital admission was 17.23 ± 6.65 months (3–81 months). Primary or secondary amenorrhea was confirmed in all the patients. The mean body weight in the AN group as of the first day of hospitalisation was 42.77 ± 2.19 kg (29.2–54.8 kg), and the mean value of their body mass index (BMI) was 15.78 ± 0.64 kg/m^2^ (11.2–18.1 kg/m^2^) (see Table 1). The BMI value, expressed as the standard deviation score (SDS) for gender and age in the girls with AN, calculated on the basis of the current centile curves for the population of Polish girls (Palczewska et al.) [8], was −2.22 ± 0.32 (−4.35 to −0.46) (see Table 1). The mean Cole’s index, reflecting the nutritional status in the AN group, was 78% ± 3% (54–95%) (Table 1). 

The characteristic features of the girls from the control groups are presented in Table 1. 

### 3.2. Blood Collection Conditions and Methods Used for Laboratory Assays 

Blood samples were collected for lab tests in all the groups between 7:00 and 8:00 a.m. after a break in food and fluid intake of at least 12 h overnight. Five millilitres of blood was collected from a venous vessel (usually the ulnar vein). The blood sampling was handled by appropriately trained nursing staff. The serum, obtained after centrifugation, was stored at a temperature of −70 °C until the assay. 

ZAG levels in blood serum were assayed by means of an enzyme immunoassay with the application of the Bio-Vendor LLC (BioVendor—Laboratorní medicína a.s., Brno, Czech Republic) test, in compliance with the manufacturer’s instructions for use (IFU). The identification of immune complexes was based on reactions with a polyclonal rabbit antibody against human ZAG, coupled with horseradish peroxidase and, subsequently, with a solution of TMB as a substrate (TMB Substrate, slow kinetic, Sigma, Asheville, NC, USA). For the purposes of determining the concentrations of the examined samples, a calibration curve was drawn, applying the standards included in the kit. Absorbance readings were conducted by means of a µQUANT Universal Microplate Spectrophotometer from BIO-TEK INC (Bio-Tek World Headquarters, El Segundo, CA, USA) with a wavelength of 620 nm, and the results were compiled using KCJunior software (Bio-Tek, Winooski, VT, USA). The kit sensitivity was 0.673 ng/mL, and the in-series and out-of-series errors were 4.7% and 6.6%, respectively.

Biochemical analyses of the concentrations of total cholesterol, HDL, LDL, triglycerides, C-reactive protein (hsCRP), glucose, insulin, TSH, fT4, and cortisol were performed by means of a Cobas apparatus. 

In all the trial subjects, the homeostasis model assessment of insulin resistance (HOMA-IR) was carried out. 

One of the limitations of the study was the small sample size, and, in consequence, the small subgroups. We also did not evaluate the serum ZAG concentration after weight gain in the study group. 

### 3.3. Approval by the Bioethics Committee

The trial was approved by the Bioethics Committee of the Medical University of Silesia in Katowice (resolution no. KNW/0022/KB1/76/14, 1 July 2014), and the written consent of the patients and their parents or legal guardians was obtained.

## 4. Statistics

A database was drawn up in a Microsoft Excel spreadsheet, and MedCalc software ver. 12.4 was used for statistical calculations, adopting the statistical significance level of α = 0.05. The following statistical parameters were taken into account and calculated: arithmetic mean, median, minimum and maximum value, lower and upper percentile, standard error (SE), and the 95% confidence interval for the mean value. 

The compliance of the distribution of all the parameters with the normal distribution was verified. In the compliance assessment, the D’Agostino–Pearson test was used, along with variable histograms, the Gaussian curve, and marked normal probability graphs. An analysis of variance (ANOVA) was carried out. The homogeneity of variance was checked by means of Levene’s test. Kruskal–Wallis one-way ANOVA non-parametric tests and tests for multiple comparisons were applied. The Spearman’s rank correlation coefficient was calculated.

## 5. Results 

The mean body weight and BMI, expressed both in absolute values and as SDSs, as well as the Cole’s index, differed in a statistically significant way in the analysed groups (*p* < 0.05). In the girls with anorexia nervosa*,* the mean body weight, BMI, BMI-SDS, and the Cole’s index were statistically significantly lower when compared to their corresponding values in healthy and obese girls (see Table 1). 

**Figure 1:**
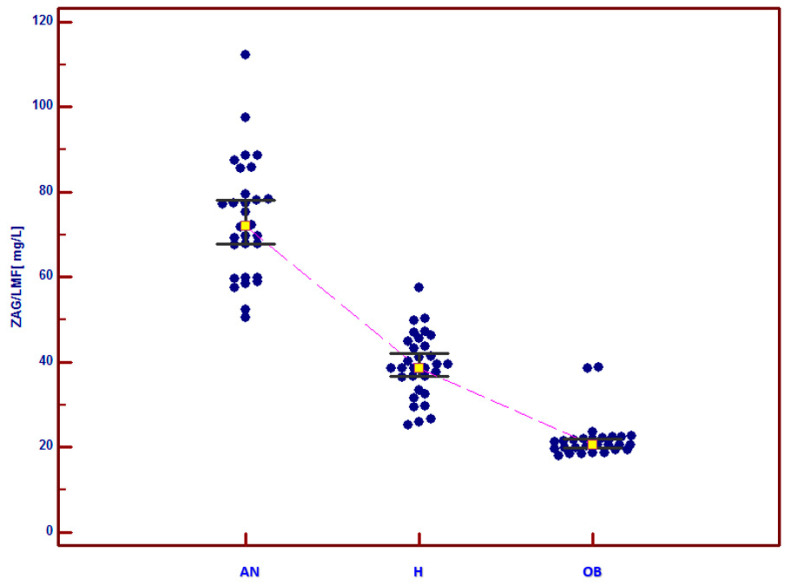
ZAG concentrations in the blood serum of the study subjects. AN—the group of subjects with anorexia nervosa, H—the group of healthy girls, OB—the group of girls with simple obesity. *p*< 0.05 AN vs. H; *p* < 0.05 AN vs. OB; *p* < 0.05 OB vs. H.

The mean concentration of ZAG in the blood serum in AN girls (73.43 ± 4.96 mg/L; range: 50.5–112.3 mg/L) was statistically significantly higher than that in the H group (39.18 ± 2.77 mg/L; range: 25.3–57.6 mg/L; *p* < 0.05) and in the OB group (21.96 ± 1.91 mg/L; range: 18.1–38.9 mg/L; *p* < 0.05). The mean ZAG concentration in the OB group was statistically significantly lower than that in the H group (*p* < 0.05) (Figure 1, see Table 2).

No significant associations were observed between illness duration and the assayed protein concentrations in the girls with AN.

Likewise, no significant correlations were demonstrated between blood serum ZAG levels and body weight, BMI, BMI-SDS, and the Cole’s index values in either the AN group or the control groups. 

However, the study demonstrated statistically significant negative correlations between ZAG concentrations in the blood and body weight (r = −0.802; *p* < 0.0001); BMI (r = −0.848; *p* < 0.0001); BMI-SDS (r = −0.848; *p* < 0.0001); and the Cole’s index (r = −0.836; *p* < 0.0001) values for the entire group of girls, analysed collectively (see Table 3). 

The mean concentrations of the analysed parameters in each group are compared in Table 4. 

The mean concentration of glucose in the AN group (78.59 ± 2.97 mg/dL; range: 66.7–104 mg/dL; *p* < 0.05) was significantly lower when compared to that in the control group of healthy girls (88.74 ± 2.8 mg/dL; range: 74–106.5 mg/dL; *p* < 0.05) and in the obese girls. Glycaemia in the group with simple obesity (84.10 ± 3.09 mg/dL; range: 72–105 mg/dL; *p* < 0.05) was significantly lower than that in the group of healthy girls (see Table 4). 

The mean concentration of insulin in the group of obese girls (27.08 ± 5.62 uU/mL; range: 4.84–66.1 uU/mL; *p* < 0.05) was significantly higher than that in the other groups (AN: 5.32 ± 1.92 uU/mL, range: 0.39–25.9 uU/mL, *p* < 0.05; H: 11.28 ± 2.28 uU/mL, range: 3.04–33.46 μU/mL, *p* < 0.05 ) (see Table 4).

The mean value of the HOMA-IR coefficient was also significantly higher in the group with obesity (4.49 ± 0.64; range: 0.954–8.389; *p* < 0.05) when compared to that in the other analysed groups. Furthermore, the value of this coefficient (0.82 ± 0.25) was statistically significantly lower (*p* < 0.05) in the patients with anorexia nervosa when compared to the group of healthy girls (2.51 ± 0.52).

No statistically significant correlations were observed between the ZAG levels and the parameters of the carbohydrate metabolism in either the group of girls with anorexia nervosa or the other analysed groups. 

However, statistically significant negative correlations were observed between the ZAG levels and insulin (r = −0.731; *p* < 0.0001) and HOMA-IR (r = −0.74; *p* < 0.0001) values for the entire group of girls, collectively analysed (see Table 5). 

The mean concentrations of the lipid metabolism parameters analysed in each group are compared in Table 6. 

The mean concentrations of total cholesterol did not differ significantly among the analysed groups of subjects. 

The mean LDL concentration was statistically significantly higher in the AN group (2.76 ± 0.37 mmol/L; range: 0.77–5.3 mmol/L; *p* < 0.05) than in the group of healthy patients (2.21 ± 0.22 mmol/L; range: 0.81–3.54 mmol/L; *p* < 0.05), while it did not differ significantly from that in the group of obese girls (2.82 ± 0.27 mmol/L; range: 1.56–4.15 mmol/L). The mean concentration of LDL in the group of obese subjects was statistically significantly higher than that in the group of healthy girls. 

The mean concentration of HDL was statistically significantly higher in the AN group (1.73 ± 0.16 mmol/L; range: 0.9–2.86 mmol/L; *p* < 0.05) when compared to the group of obese girls (1.05 ± 0.12 mmol/L; range: 0.59–1.94 mmol/L; *p* < 0.05), while it did not differ significantly from that in the group of healthy subjects (1.54 ± 0.15 mmol/L; range: 0.71–2.61 mmol/L; *p* < 0.05). The mean concentration of HDL in the group of obese girls was statistically significantly lower than in the other groups. 

The mean concentration of triglycerides in the group of girls with obesity (1.29 ± 0.18 mmol/L; range: 0.33–2.72 mmol/L) was significantly higher than in the other groups (AN: 1.06 ± 0.34 mmol/L, range: 0.35–5.85 mmol/L, *p* < 0.05; H: 0.9 ± 0.18 mmol/L, range: 0.4–3.07 mmol/L, *p* < 0.05). The mean concentrations of triglycerides did not differ significantly between the group with anorexia nervosa and the group of healthy girls. 

No statistically significant correlations were observed between the ZAG levels and the lipid metabolism parameters in the AN, H, and OB groups. 

However, for the entire analysed group, a statistically significant positive correlation was observed between the ZAG concentrations and HDL (r = 0.514; *p* < 0.0001), and a negative correlation was found with the level of triglycerides in the blood (r = −0.278; *p* = 0.009) (Table 7).

The mean concentrations of the analysed hormones in each group of the study subjects are presented in Table 8.

No statistically significant differences were observed between the TSH concentrations in the groups of analysed girls. 

The mean concentration of FT4 in the AN group (1.06 ± 0.07 ng/dL; range: 0.728–1.5 ng/dL; *p* < 0.05) was statistically significantly lower than that in the H group (1.21 ± 0.06 ng/dL; range: 0.954–1.73 ng/dL; *p* < 0.05), and it did not significantly differ from that in the group of obese girls (1.15 ± 0.07 ng/dL, range: 0.964–1.63 ng/dL). 

The mean concentration of cortisol was significantly higher in the AN group (20.6 ± 2.07 ug/dL; range: 10.28–39.63 ug/dL) than in the other examined groups of girls (H: 15.09 ± 2.22 ug/mL, range 3.81–28.99 ug/mL, *p* < 0.05; OB: 13.88 ± 1.48 ug/dL, range: 7.56–19.37 ug/dL, *p* < 0.05).

A statistically significant positive correlation was observed between the ZAG concentrations and blood cortisol levels (r = 0.482; *p* < 0.0001) (see Table 9) for the entire analysed group of patients.

The mean concentration of hsCRP in the blood serum of the AN patients (0.99 ± 0.61; range: 0.05–4.33 mg/L) was significantly lower than that in the obese girls (2.57 ± 1.34 mg/L; range: 0.38–14.68 mg/L; *p* < 0.05), and it did not significantly differ from the corresponding value in the group of healthy girls (0.59 ± 0.31 mg/L; range: 0.02–4.17 mg/L; *p* < 0.05). The concentration of hsCRP was not elevated in any of the patients in either the AN group or the H group. On the other hand, the concentration of hsCRP in the OB group was beyond its normal limits (N: 0–5 mg/L). Furthermore, it was demonstrated that the mean concentration of hsCRP in the obese girls was statistically significantly higher than that in the healthy subjects (*p* < 0.05).

No statistically significant correlations were observed between the concentrations of ZAG and hsCRP in each group of patients.

On the other hand, the ZAG concentration negatively correlated with the concentration of hsCRP (r = −0.367, *p* = 0.002) for the entire group of study subjects. 

### Analysis of Results and Discussion

Divergent data are found in reports on immunological system disorders observed in the course of anorexia nervosa. Some researchers suggest their key contribution to the pathogenesis of the disease, whereas others point merely to certain irregularities [9,10,11]. Further research into this issue is encouraged by studies of other mental disorders, such as depression or schizophrenia, in which considerable differences have been demonstrated in the composition and concentrations of individual elements of the immunological system, particularly proinflammatory cytokines, as compared to healthy individuals [12,13,14].

Our study demonstrated a significantly higher concentration of ZAG in the blood serum in the analysed group of female patients with anorexia nervosa when compared to the groups of healthy and obese girls. The concentrations of ZAG in the blood serum of the obese girls were also significantly lower than that in the other two groups. Similar results were also obtained by other researchers [15,16,17,18]. There are also studies that did not demonstrate any significant differences between the blood concentrations of ZAG in patients with a normal body mass versus obese individuals [4,19]. 

Selva et al. [15] conducted a study in a group of 20 obese adult patients (BMI 42.8 ± 2.5 kg/m^2^) subjected to bariatric surgery and 10 individuals with a normal body weight after cholecystectomy, who constituted a control group (BMI 25.1 ± 3.1 kg/m^2^). In all the study subjects, serum ZAG concentrations were evaluated, along with the expression of ZAG (ZAG mRNA and protein) in the subcutaneous (SAT) and visceral adipose tissue (VAT), as well as in material from liver biopsies. It was demonstrated, similarly to our study, that the ZAG concentration in the blood serum in obese patients was significantly lower than that in the control group (40.87 ± 10.45 vs. 63.26 ± 16.40; *p* = 0.002). Furthermore, a significantly lower expression of ZAG in SAT, VAT, and liver biopsy material was demonstrated in the obese subjects. Additionally, the ZAG concentration in the blood serum exhibited a significant negative correlation with BMI across the entire analysed group (r = −0.65; *p* < 0.001). 

This correlation across the entire analysed group, in which the spread of body mass values was fairly broad, was also demonstrated in our study. A similar relationship was observed by the researchers in relation to the ZAG expression in SAT and VAT. The authors cited did not demonstrate any significant associations between ZAG concentrations and the insulin resistance factor (HOMA-IR). This was a divergent result compared to that obtained in our study, where we confirmed a negative correlation between the ZAG concentrations on the one hand and the insulin concentration and HOMA-IR factor on the other. Perhaps this difference resulted from the higher number of subjects in our study and from the fact that it also involved female patients with anorexia nervosa, in whom this factor usually demonstrates the lowest values. 

Results coinciding with ours were obtained by Yang et al. in their study [17] in a group of 85 patients with abnormal glucose tolerance and 100 patients with de novo type II diabetes compared to a control group of 100 healthy individuals. They demonstrated that the concentration of ZAG in the blood serum in the analysed groups was statistically significantly lower than that in the control group. Furthermore, they proved a negative correlation between the ZAG concentrations in the blood serum and BMI; insulin concentration; HOMA-IR; and the concentration of triglycerides, fasting glucose, and HbA1c. This observation may suggest that, besides the lipolysis-intensifying activity, ZAG also exerts its effects on the insulin sensitivity of tissues. 

Barraco et al. [5] explored the relationship between ZAG and insulin sensitivity within a population of children. In their study, they examined 303 patients, divided into two groups: 138 children with a normal body weight and 165 children with overweight/obesity. The mean age was 5.49 ± 0.09 (range: 2–7.9 years). In children with overweight/obesity, the ZAG concentrations in the blood serum were statistically significantly lower than those in healthy children. Subsequently, the subjects were divided into the following age groups: 2–4 years, 5–6 years, and 7–8 years. It was demonstrated that in children with a normal body weight, the concentration of ZAG exhibited a growing tendency with age, which was not observed in children with overweight/obesity. No sex-related differences were confirmed, either. The ZAG concentrations correlated negatively with insulin resistance, expressed by the HOMA-IR factor in both analysed groups, as well as with the fasting insulin concentration and BMI. Our results were considerably similar to those provided in the study cited above. Therefore, it can be concluded that ZAG is a molecule that most probably participates in the process of insulin sensitivity modelling as early as childhood [5].

The ZAG molecule is known to be produced by—without limitations—adipocytes and to demonstrate the adipocytokine effect. There are increasing numbers of reports relating to the paraendocrine role of ZAG within adipose tissue. 

In several previously conducted studies, a negative correlation was observed between the ZAG gene expression and the insulin concentration in the serum [16,20]. This fact can be accounted for by the intensification of adiponectin expression by ZAG in adipose cells, which is closely connected with insulin resistance control [21,22].

Garrido-Sanchez et al. [23] examined this issue more closely, developing a study with the participation of 25 patients with morbid obesity (BMI 57.4 ± 5.2 kg/m^2^), divided into two groups in terms of insulin sensitivity. The first group consisted of 11 patients with a low insulin resistance factor (HOMA-IR 2.85 ± 0.734), whereas the other group comprised 14 patients with a high value of this factor (HOMA-IR 11.46 ± 4.73). The study exclusively covered patients without diagnosed diabetes. The concentrations of ZAG in the blood plasma and its expression in subcutaneous and visceral adipose tissue (SAT and VAT) were analysed. It was demonstrated that the patients with low insulin resistance in their tissues had a significantly higher ZAG expression level within the visceral adipose tissue when compared to the patients with high insulin resistance (*p* = 0.023). Such a relationship was not demonstrated, however, for subcutaneous fat tissue. Serum ZAG concentrations, in contrast to our study, did not differ significantly between the groups. This observation may suggest a very significant paraendocrine regulation of this protein related to insulin resistance modelling. 

Apart from the study cited above, other authors have not demonstrated any significant differences in ZAG concentrations in the blood serum among healthy or obese patients. 

Stejskal et al. [4] did not observe any differences in ZAG concentrations in the blood serum between healthy subjects and patients suffering from metabolic syndrome. Balaz et al. [19] demonstrated a reduced ZAG expression level in subcutaneous adipose tissue in obese subjects with a tendency towards a further reduction in pre-diabetic conditions and in patients with type 2 diabetes. However, they did not observe any significant differences in ZAG concentrations in the analysed groups. 

To summarise, there are numerous data pointing to reduced ZAG expression levels, predominantly in subcutaneous adipose tissue (SAT), in patients with obesity, plus variable data relating to blood serum concentrations. ZAG seems to act as an adipocytokine involved in the process of modelling the insulin sensitivity of tissues. 

Among the studies assessing the role of ZAG as a molecule involved in the pathogenesis of cachexia, studies carried out on a population of patients with cancer are the most numerous. These studies initially led to isolating this molecule from the urine in patients experiencing the neoplastic process, and they still constitute the fundamental source of information on ZAG. So far, increased concentrations of ZAG have been demonstrated in the secretory epithelium of cancers such as liver cancer, breast cancer, prostate cancer, and gastrointestinal cancer [24]. The development of neoplastic cachexia is triggered by the loss in body fat mass and—according to in vitro and in vivo studies carried out on animal models—the intensification of energy expenditure, for which the intensification of the expression of uncoupling proteins (UCPs) is responsible [25,26,27]. 

The relationship between ZAG and the expression of uncoupling proteins (UCPs) was confirmed by Bing et al. in their animal study [26]. They selected a group of six mice, which were injected with ZAG isolated from the urine of cachectic patients suffering from cancer. After the intravenous administration of this protein, repeated several times, the mice were decapitated, and their serum and tissues were sampled for further examination. It was proven that ZAG caused a quick loss in body fat mass in mice (by 10% when compared to controls after 52 h, *p* = 0.03) without any significant loss in muscle mass. This drop was not associated with a decreased appetite in the animals. Furthermore, the analysed mice exhibited a significantly lower concentration of leptin in the blood serum, proportional to the loss in body weight (*p* < 0.01). The study also examined the expression of the uncoupling proteins UCP-1, UCP-2, and UCP-3 in the adipose tissue sampled from the scapula region, detecting higher expression in the analysed animals (UCP-1 +96%, *p* < 0.01; UCP-2 +57%, *p* = 0.02; UCP-3 +37%, *p* < 0.05). Furthermore, a higher expression of UCP-2 (+142%, *p* = 0.03) was demonstrated in the livers of the studied mice. The histopathology of all the liver lobes demonstrated the presence of numerous microdrops of lipids, which were not observed in the same quantities in the control group. On the basis of this research, the authors suggested that the intensified expression of UCPs facilitates the metabolic use of lipid excess, which occurs in the process of progressive, ZAG-induced cachexia.

Cancer cachexia is associated with a progressive loss in fat and lean body mass. Patients who demonstrate this phenomenon have an unfavourable prognosis in terms of life expectancy. The development of cancer cachexia is influenced by numerous immune factors, secreted by both tumour tissues and host cells. Some immunological and metabolic parallels to mental anorexia nervosa have been sought in the observation of cancer patients with anorexia–cachexia syndrome. These patients, especially those with advanced stages of cancer, significantly restrict their food intake. This eating disorder may be caused by a preponderance of appetite-inhibitory signals in the hypothalamus (proopiomelanocortin and pro-inflammatory cytokines IL-1α, IL-1β, IL-6, TNF-α, or MIC-1). It is worth mentioning that in the case of anorexia nervosa, there is a similar predominance of the aforementioned cytokines and neuromodulators in this part of the brain. Metabolic changes involving an increase in resting energy expenditure and disturbances in carbohydrate, protein, and lipid metabolism are also associated with cancer cachexia. From this perspective, the role of the protein we studied, namely α2-glycoprotein zinc, which enhances lipolysis, also seems a highly interesting [6]. No research project has been conducted to determine whether high ZAG concentrations in the blood serum of AN patients constitute a mechanism that is also intended to make use of the products of lipid catabolism, or whether they constitute a causative agent triggering the body weight loss process. 

In light of the research carried out in both patients with cancer and animal models, it seems that high ZAG levels do not affect the appetite; thus, seeking the primary causes of anorexia nervosa in ZAG concentrations would be an approach detached from the available evidence. However, ZAG may indicate a secondary contributor towards progressive cachexia caused by lower food intake. The anorectic girls included in our study had reported at least several months of a deliberate reduction in food intake; hence, the high concentrations of ZAG in their blood serum may have resulted from these behaviours, which, in effect, led to intensified lipolysis and accelerated the rate of their body weight loss processes. 

The high ZAG concentrations in the analysed group could also have been caused by hormonal disorders in relation to—without limitations—the hypothalamic–pituitary–adrenal axis. In our study, we demonstrated a significantly higher concentration of cortisol in the blood serum in the girls with anorexia nervosa compared to the other groups. 

A relationship between glycocorticosteroids and ZAG was proven by Russel and Tisdale in their study [28]. They demonstrated that dexamethasone administered to healthy experimental animals caused a six-fold increase in the expression of ZAG in both white and brown adipose tissue (WAT and BAT). A similar effect was caused by dexamethasone in a culture of human adipocytes. In both cases, its administration brought about intensified lipolysis. An additional application of the antagonist of the glycocorticoid receptor annulled this effect. A similar result was obtained after the application of an anti-ZAG antibody and the antagonist of the *β*3-adrenergic receptor.

In our project, we also demonstrated a positive correlation between cortisol concentrations and zinc-alpha 2 glycoprotein (r = 0.482, *p* < 0.0001) levels, analysed collectively across the entire study group. 

Therefore, considering the fact that in both patients with anorexia nervosa and those with cancer, the cortisol concentrations in the blood serum were elevated and correlated positively with body weight loss (also supported by the mice model of cachexia in our study), it can be concluded that the cachectic effect of glycocorticosteroids may be caused by increased ZAG concentrations and their lipolytic effects. 

In our study, we also demonstrated a negative correlation between blood concentrations of ZAG and hsCRP levels across the entire study group of girls, while no such correlation was demonstrated within individual groups. The obese girls exhibited statistically significant hsCRP concentrations in the serum compared to the anorectic and healthy girls. The C-reactive protein is an acute-phase protein produced in the liver through the stimulating effect of interleukin 6. The method of assaying this protein (*high-sensitivity CRP* (hsCRP)) adopted in our study allowed us to analyse low concentrations, undetectable by routine methods. We proved an association between the concentrations of hsCRP and the incidence of cardiovascular diseases. A concentration of hsCRP above 2 mg/L in the blood serum constitutes a significant risk factor for the development of such conditions. In our study, the mean concentrations of this protein in the blood serum of the obese girls oscillated around 2.5 mg/L; therefore, these girls were more exposed to the risk of cardiovascular incidents in the future than the other patients (AN group: 0.99 mg/L, H group: 0.59 mg/L) [29]. 

Some studies have demonstrated elevated concentrations of hsCRP in patients with obesity, which could be a manifestation of a low-intensity generalised inflammatory process [29,30,31]. Furthermore, the concentration of hsCRP drops both after body mass reduction and during physical activity. Patients with anorexia nervosa very often admit that they exercise intensely, which may additionally contribute to the low concentrations of this protein in this group of patients. 

The relationship between ZAG and the acute-phase protein hsCRP has not yet been fully explored. Some studies indicate that ZAG does not participate in inflammation-inducing processes in adipose tissue. In vitro studies on cultures of human adipocytes have demonstrated that ZAG expression is reduced under the influence of TNF-*α*, which is a cytokine whose close relationship to low-intensity inflammation has been proven in obesity. The final influence of ZAG and TNF-*α* on adipose tissue, however, is convergent—both proteins induce lipolysis, although through mechanisms specific to each. On the other hand, the effect of IL-6 on the expression of ZAG has not yet been detected [32,33]. 

Also of note in the hormone assay results obtained herein were the statistically significantly lower FT4 levels in the anorexia group compared to the healthy and obese girls. According to literature reports, these patients show not only decreased T4 levels but also the decreased conversion of T4 to T3 and the increased conversion of FT4 to the metabolically inactive form of T3 (reverse T3 (rT3)). This is due to the desire of a body subjected to chronic malnutrition to reduce its resting energy expenditure and slow the rate of further weight loss [34]. As the body weight increases, the total T3 concentration increases, and the rT3 concentration decreases [35].

In summary, our research indicated that ZAG may play a role in individuals with eating disorders. We demonstrated significantly higher concentrations of this substance in the blood of the patients suffering from anorexia nervosa when compared to healthy and obese girls. 

Taking into account the effect of ZAG, intensifying the breakdown of lipids, this protein may be considered an anchor point in possible future therapy models of anorexia nervosa. 

## 6. Conclusions

1. Girls with anorexia nervosa differed from healthy and obese girls due to the significantly higher concentrations of ZAG in their blood.
2. The high concentrations of ZAG in the blood of the patients with anorexia nervosa seemed to constitute an adaptation mechanism, secondary to undernourishment, facilitating the use of lipolysis products for the patient’s system.
3. The ZAG concentrations in the blood of the girls with anorexia nervosa did not depend on the illness duration. They did, however, correlate negatively with the parameters of nutritional status (body mass, BMI, and the Cole’s index), which was collectively observed across the entire study group in our research project.
4. The elevated concentrations of ZAG in the blood of the patients with anorexia nervosa may have resulted from an elevated concentration of cortisol in the blood. This is a manifestation of the incorrect functioning of the hypothalamic–pituitary–adrenal axis in such patients that may additionally promote body weight loss.
5. The elevated serum ZAG levels observed in patients with mental anorexia nervosa, as in patients with cancer, may have been a phenomenon secondary to malnutrition caused by an underlying disease.

## Figures and Tables

**Table 1:** Characteristic features of the examined girls.

	AN n = 30	H n = 30	OB n = 27
	Mean ± 1.96 SEM (Range)	Mean ± 1.96 SEM (Range)	Mean ± 1.96 SEM (Range)
Age (y)	15.64 ± 0.41 (13.1–17.3)	16.17 ± 0.54 (11.2–17.9)	13.73 ± 0.82 (11.0–17.0)
Body weight (kg)	42.77 ± 2.19 ^ac^ (29.2–54.8)	54.43 ± 3.01 (39–73.6)	90.15 ± 6.78 ^b^ (54.0–118.8)
BMI (kg/m^2^)	15.78 ± 0.64 ^ac^ (11.2–18.1)	19.92 ± 0.8 (16–24)	34.47 ± 1.94 ^b^ (26.1–43.57)
BMI-SDS	−2.22 ± 0.32 ^ac^ (−4.35–−0.46)	−0.14 ± 0.45 (−2.72–1.94)	6.88 ± 1.0 ^b^ (3.24–12.87)
Cole index (%)	78 ± 3 ^ac^ (54–95)	98 ± 4 (78–116)	181 ± 10 ^b^ (127–253)
Body height (cm)	164.22 ± 2.64 (151–182.3)	164.98 ± 2.33 (149.9–177)	161.21 ± 3.24 (135–173)
Maximum body weight prior to the disease (kg)	58.98 ± 4.35 (36.0–82.0)	_	_
Illness duration (months)	17.23 ± 6.65 (3–81)	_	_
Body weight loss (kg)	15.92 ± 2.77 (5.3–33.7)	_	_

AN—the group of subjects with anorexia nervosa, H—the group of healthy girls, OB—the group of girls with simple obesity. ^a^ *p* < 0.05 AN vs. H; ^b^ *p* < 0.05 SO vs. H; ^c^ *p* < 0.05 AN vs. SO.

**Table 2:** Mean blood serum ZAG concentrations in each study group.

	AN	H	OB
	Mean ± 1.96 SEM (Range)	Mean ± 1.96 SEM (Range)	Mean ± 1.96 SEM (Range)
ZAG (mg/L)	73.43 ± 4.96 ^ac^ (50.5–112.3)	39.18 ± 2.77 (25.3–57.6)	21.96 ± 1.91 ^b^ (18.1–38.9)

AN—the group of subjects with anorexia nervosa, H—the group of healthy girls, OB—the group of girls with simple obesity. ^a^ *p* < 0.05 AN vs. H; ^b^ *p* < 0.05 OB vs. H; ^c^ *p* < 0.05 AN vs. OB.

**Table 3:** Correlations between blood ZAG levels and body weight, BMI, BMI-SDS, and the Cole’s index values for the whole study group.

	Whole Study Group
	ZAG (mg/L)
Body weight (kg)	r = −0.802 *p* < 0.0001
BMI (kg/m^2^)	r = −0.848 *p* < 0.0001
BMI-SDS	r = −0.828 *p* < 0.0001
Cole’s index (%)	r = −0.836 *p* < 0.0001

**Table 4:** Mean concentrations of glucose, insulin, and HOMA-IR coefficient values in the analysed groups.

	AN	H	OB
	Mean ± 1.96 SEM (Range)	Mean ± 1.96 SEM (Range)	Mean ± 1.96 SEM (Range)
Glucose (mg/dL) (norm (N): 60–100 mg/dL)	78.59 ± 2.97 ^ac^ (66.7–104)	88.74 ± 2.8 (74–106.5)	84.10 ± 3.09 ^b^ (72–105)
Insulin (uU/mL) (N: 2.6–24.9 uU/mL)	5.32 ± 1.92 (0.39–25.9)	11.28 ± 2.28 (3.04–33.46)	27.08 ± 5.62 ^d^ (4.84–66.1)
HOMA-IR (N: < 2)	0.82 ± 0.25 ^ac^ (0.07–3.77)	2.51 ± 0.52 (0.599–7.421)	4.49 ± 0.64 ^b^ (0.954–8.389)

AN—the group of subjects with anorexia nervosa, H—the group of healthy girls, OB—the group of girls with simple obesity. ^a^ *p* < 0.05 AN vs. H; ^b^ *p* < 0.05 OB vs. H; ^c^ *p* < 0.05 AN vs. OB; ^d^ *p* < 0.05 OB vs. AN and H.

**Table 5:** Correlations between blood ZAG concentrations and the parameters of carbohydrate metabolism for the whole study group.

	Whole Study Group
	ZAG (mg/L)
Glucose (mg/dL)	r = −0.188 *p* = 0.085
Insulin (uU/mL)	r = −0.731 *p* < 0.0001
HOMA-IR	r = −0.74 *p* < 0.0001

**Table 6:** The mean concentrations of total cholesterol, LDL, HDL, and triglycerides in the study groups.

	AN	H	OB
	Mean ± 1.96 SEM (Range)	Mean ± 1.96 SEM (Range)	Mean ± 1.96 SEM (Range)
Total cholesterol (mmol/L) (N: 0–5.2 mmol/L)	4.96 ± 0.46 (2.43–7.78)	4.16 ± 0.32 (1.92–6.13)	4.45 ± 0.28 (2.91–5.68)
LDL (mmol/L) (N: < 2.59 mmol/L)	2.76 ± 0.37 ^a^ (0.77–5.3)	2.21 ± 0.22 (0.81–3.54)	2.82 ± 0.27 ^b^ (1.56–4.15)
HDL (mmol/L) (N: 1.15–1.68 mmol/L)	1.73 ± 0.16 ^c^ (0.9–2.86)	1.54 ± 0.15 (0.71–2.61)	1.05 ± 0.12 ^d^ (0.59–1.94)
Triglycerides (mmol/L) (N: 0.4–1.8 mmol/L)	1.06 ± 0.34 (0.35–5.85)	0.9 ± 0.18 (0.4–3.07)	1.29 ± 0.18 ^d^ (0.33–2.72)

AN—the group of subjects with anorexia nervosa, H—the group of healthy girls, OB—the group of girls with simple obesity. ^a^ *p* < 0.05 AN vs. H; ^b^ *p* < 0.05 OB vs. H; ^c^ *p* < 0.05 AN vs. OB; ^d^ *p* < 0.05 OB vs. AN and H.

**Table 7:** Correlations between blood ZAG concentrations and lipid metabolism parameters for the whole study group.

	Whole Study Group
	ZAG (mg/L)
Total cholesterol (mmol/L)	r = 0.148 *p* = 0.169
LDL (mmol/L)	r = −0.023 *p* = 0.835
HDL (mmol/L)	r = 0.514 *p* < 0.0001
TG (mmol/L)	r = −0.278 *p* = 0.009

**Table 8:** The mean concentrations of TSH, FT4, and cortisol in the blood serum of the study subjects.

	AN	H	OB
	Mean ± 1.96 SEM (Range)	Mean ± 1.96 SEM (Range)	Mean ± 1.96 SEM (Range)
TSH (ulU/mL) (N: 0.27–4.2 uIU/mL)	2.08 ± 0.43 (0.96–5.5)	2.15 ± 0.31 (0.806–4.42)	2.28 ± 0.32 (1.11–4.18)
FT4 (ng/dL) (N: 0.93–1.7 ng/dL)	1.06 ± 0.07 ^a^ (0.728–1.5)	1.21 ± 0.06 (0.954–1.73)	1.15 ± 0.07 (0.964–1.63)
Cortisol (ug/dL) (N: 2.3–19.4 ug/dL)	20.6 ± 2.07 ^b^ (10.28–39.63)	15.09 ± 2.22 (3.81–28.99)	13.88 ± 1.48 (7.56–19.37)

AN—the group of subjects with anorexia nervosa, H—the group of healthy girls, OB—the group of girls with simple obesity. ^a^ *p* < 0.05 AN vs. H; ^b^ *p* < 0.05 AN vs. H and OB.

**Table 9:** Correlations between ZAG and tested hormone concentrations in the blood for the whole study group.

	Whole Study Group
	ZAG (mg/L)
TSH (ulU/mL)	r = −0.15 *p* = 0.176
FT4 (ng/dL)	r = −0.115 *p* = 0.296
Cortisol (ug/dL)	r = 0.482 *p* < 0.0001

## Data Availability

The datasets generated during and/or analyzed during this study are available from the corresponding author upon reasonable request.
